# The Biology and some Population Parameters of the Grasshopper, *Ronderosia bergi*, Under Laboratory Conditions

**DOI:** 10.1673/031.010.9201

**Published:** 2010-07-05

**Authors:** Yanina Mariottini, Maria Laura de Wysiecki, Carlos Lange

**Affiliations:** ^1^ Centro de Estudios Parasitológicos y de Vectores (CEPAVE - CCT La Plata CONICET-UNLP), La Plata, Argentina; ^2^ Comisión de Investigaciones Científicas (CIC), Buenos Aires province, Argentina

**Keywords:** Acrididae, cohorts, demography, fecundity, longevity

## Abstract

Some biological and population parameters of *Ronderosia bergi* (Stål) (Orthoptera: Acrididae: Melanoplinae) were estimated by monitoring five cohorts of the first generation (F1) of individuals born in captivity from grasshoppers collected in the South of Misiones province, northeastern Argentina, and held under controlled conditions (30° C, 14:10 L:D, 40% RH). The mean embryonic development time was 40.6 ± 1.7 days. Five nymphal instars were recorded. Total duration of nymphal development was 30.8 ± 0.54 days. The mean lifespan of cohorts was 22.6 ± 0.7 weeks. The number of egg-pods per female was 7.6 ± 1.44, and the amount of eggs per egg-pod was 16.45 ± 0.85. Mean fecundity was 125 ± 5.83 eggs per female with an oviposition rate of 1.55 ± 0.57 eggs/female/day. Survivorship curves showed that mortality was concentrated in the final weeks of adulthood, and the life expectancy curve decreased accordingly. The population parameters estimated gave the following values: the net rate of reproduction (R_0_) was 46.75 ± 11.2, generation time (T) was 18.87 ± 1.67 weeks, duplication time (D) was 3.31 ± 0.34, the intrinsic rate of population growth (r_m_) was 0.21 ± 0.021 and the finite rate of population increase (λ) was 1.24 ± 0.026. The reproductive values (V_x_) indicated that the largest contribution of females to the subsequent generation was between weeks 15 and 25.

## Introduction

The South American genus of melanopline grasshoppers *Ronderosia* is known to occur in the Chacoan and Amazonian biogeographic regions, mainly in the Cerrado and Paranaense biogeographic provinces (Cigliano 1997). *Ronderosia bergi* (Stål) (Orthoptera: Acrididae: Melanoplinae) has the widest geographic distribution of the genus, embracing central and north Argentina, Paraguay, southeastern Bolivia, southern Brazil, and Uruguay (Lange et al. 2005). It is a common species in natural grasslands and a variety of crops including alfalfa, sunflower, tobacco, corn, and others (Carbonell et al. 2006). It has been included as one of 15 grasshopper species of economic importance in Argentina (Lange et al. 2005) due to damage historically reported from different areas of the country (Ronderos 1959; Liebermann 1972; COPR 1982). Following the categories most widely accepted for defining the pest status of grasshopper species (COPR 1982), Carbonell et al. (2006) have categorized *R. bergi* as an occasional pest of local importance. Historically, Liebermann (1941, 1948) has recorded high densities (i.e., outbreaks, 25 ind/m^2^) in northeastern Argentina. Likewise, the fact that *R. bergi* lacks an obligatory embryonic diapause and it is easy to handle due to its little reactive behavior makes it an amenable species for the development and maintenance of breeding colonies in captivity under controlled conditions, which in turn are useful for conducting experimental studies with biocontrol agents (Lange 2003, 2004; Lange and Cigliano 2004). Another salient characteristic of *R. bergi* is its notorious intraspecific morphological variability that over the years has even led to the designation of different species, subspecies, and geographic varieties (Cigliano 1997; Carbonell et al. 2006). Mariottini et al. (2006) have studied some biological attributes of the post-embryonic development of *R. bergi* under controlled conditions using laboratory-reared individuals whose geographic origin was San Luis province in central Argentina, close to the south western end of the species range (Lange et al. 2005). According to Southwood (1994), to determine the demographic parameters and vital statistics of pest species from laboratory life tables constitutes a basic initial tool for the development of control strategies. Additionally, in order to detect and evaluate any possible differences in the biological parameters of the post-embryonic development of *R. bergi* in relation to the geographical origin of the specimens, a comparable study to the one reported by Mariottini et al. (2006), but with individuals collected in Misiones province at the north eastern part of the range of *R. bergi*, was conducted.

## Materials and Methods

Grasshoppers were collected as adults in fields on the surroundings of Alba Posse (27° 34′ S, 54° 41′ W), at the north eastern province of Misiones in Argentina, in March of 2006. Once in the laboratory, the insects were kept following general procedures as described by Henry (1985) for the long-term maintenance and breeding of grasshoppers in captivity. Individuals of both sexes were placed in wire-screened, aluminium cages (20 × 20 × 30 cm) in a rearing room under controlled conditions (30° C, 14:10 L:D, 40% RH) routinely used worldwide (Henry 1985; Hinks and Erlandson 1994). The insects were fed with thoroughly washed, fresh leaves of lettuce, cabbage, a variety of wild grasses, and flakes of wheat bran. Each cage was provided with a substrate for egg-pod laying that consisted of a plastic container (10 cm deep) filled with sterilized sand. Mating and thermoregulation was stimulated with 75W bulbs suspended 15 cm above each cage. Egg-pods were incubated *in situ* under the same controlled conditions. Some egg-pods (n = 20) were dismembered and examined to determine the average number of eggs per pod.

The population parameters were determined by monitoring five cohorts of 18, 22, 14, 16, and 16 individuals respectively. Embryonic development time was estimated for each cohort from egg-pod lying until hatching. The nymphal development of each cohort was monitored inside acetate tubes with wire-screened ends (50 cm long, 9 cm diameter) (Henry 1985) until they reached the fifth instar, when they were transferred to individual aluminium cages (12 × 12 × 16 cm). From each cohort, four couples (one male, one female; 20 couples total) were monitored.

Cohort survivorship (proportion of individuals surviving from birth to age x), l_x_, was estimated daily and fecundity (mean number of female progeny produced per female of age x), m_x_, was estimated at weekly intervals. Values of m_x_ were corrected by the sex proportion of each cohort (Rabinovich 1980). Sex ratio was calculated following Carey (1993) (SR= number of males, n_m_/ numbers of females, n_f_). Age-specific survival (l_x_) curves were constructed taking into account both males and females of each cohort, and fecundity (m'_x_) curves were elaborated using the female portion of the cohort. Time unit for age classes was one week.

In order to estimate population parameters the methodology developed by Rabinovich (1980) and Carey (1993) was followed.

l_x_ = Proportion of surviving to age x.

m_x_ = mean number of eggs per female of x age.

m'_x_ = mean number of eggs female per female of x age, corrected by the sex proportion.

The intrinsic rate of population increase, r_m_, that expresses the innate capability of increase in number per time unit, was calculated by Newton's method (Hadeler 1982) in Lotka's equation:


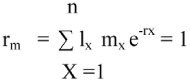


Net reproductive rate, R_0_, mean number of female offspring produced per female per generation, was calculated as:


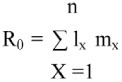


Where 1 is the age of first reproduction, and n is the age of the last reproduction (observed values).

Generation time, T, is the mean longevity of a generation (average time between two successive generations) and was calculated as:


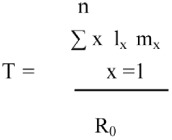


Duplication time D (number of units of time required for the population to doubling in number) was calculated as:


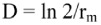


Reproductive values V_x_ (the contribution to the future population that female of age *x* will make) was calculated as:


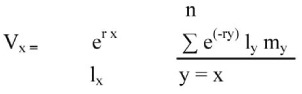


Finite rate of increase, λ, that is the number of times the population increases per time unit, was calculated as:


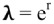


Life expectancy (e_x_), mean time is left for an individual to live according to their age within the population.


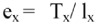


T_x_ = total number of days left to live to the individuals who have attained the age x.

Results are expressed as mean values ± standard error (SE).

A two-way ANOVA test was used in order to analyze the variability in longevity of adult individuals among cohorts and gender (males and females).

**Table 1.**
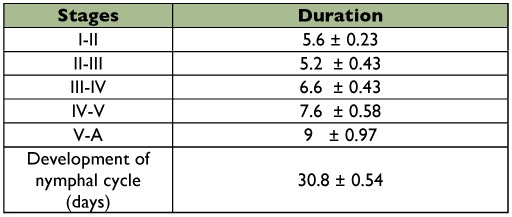
Number and duration in days (mean ± SE) of nymphal instars of *Ronderosia bergi* at 30° C and 14:10 LD photoperiod.

In order to compare the longevity of adults with the results of individuals of San Luis province (Mariottinni et al. 2006), the nonparametric test of Kruskal Wallis of ANOVA by ranks was employed. This was so because a previous test on variance homogeneity provided a significant result, not allowing the use of a parametric test. Statistical tests were performed using the XLSTAT 7.5.3 program.

## Results

Mean embryonic development time was 40.6 ± 1.7 days. Five nymphal instars were registered for both females and males. The developmental time of the nymphal cycle was 30.8 ± 0.54 days, with a minimum and a maximum of 28 and 36 days, respectively (Table 1). A proportion of 88.1 ± 1.52% of the nymphs reached the adult state. The mean sex ratio in the five cohorts was 0.503 M: 0.497 F. There was no significant difference in the amount of females and males in the cohorts (Levene: F = 0.000, g.l = 1.8 p = 1. T de Student: t = -0.154, g.l = 8 p = 0.881).

The total duration of cohorts was 22.6 ± 0.7 weeks, with a minimum of 19 weeks and a maximum of 27 weeks. The first mating, taken as an indicator of individuals reaching sexual maturity, was observed four to five days after entering adulthood. The averaged number of egg-pods per female was 7.6 ± 1.44 with a minimum of two and a maximum of 15. The number of eggs per pod was 16.45 ± 0.85, and ranged from 10 to 22. Mean fecundity (number of eggs/female) was of 125 eggs ± 5.83, with a maximum of 247 eggs and a minimum of 33. Fecundity rate was of 1.55 ± 0.57 eggs/female/days.

**Figure 1.**
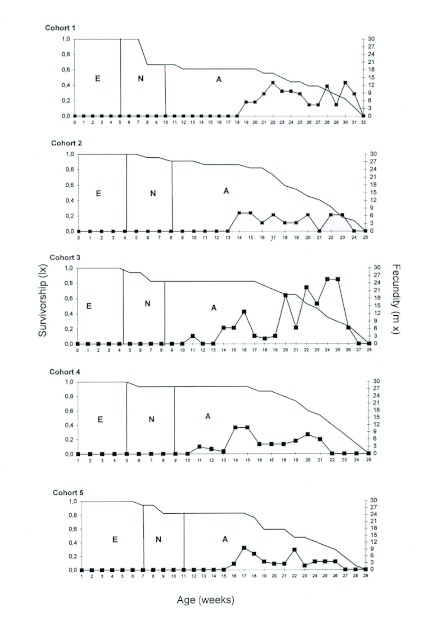
Age-specific survival (l_x_) and fecundity (m'_x_) curves of *Ronderosia bergi* at 30°C and 14:10 (L: D) photoperiod. Time unit in weeks. High quality figures are available online.

The survivorship curves showed a consistent trend of high survival during most of the development of the cohort with the bulk of mortality towards the end of the cycle (Figure 1). The fecundity curves obtained for the different cohorts (Figure 1) showed great variability, Cohort number three showed the highest values of m'_x_, particularly at weeks 24 and 25. The mean m'_x_ for all five cohorts was highest between weeks 20 to 25, reaching the top value at week 22 (9.77 ± 5.63).

**Figure 2.**
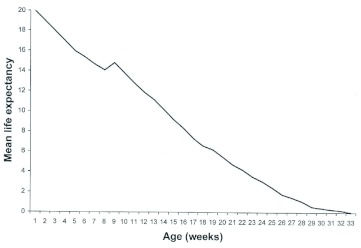
Mean life expectancy for *Ronderosia bergi*, at 30° C and 14:10 L:D photoperiod. Unit time in weeks. High quality figures are available online.

**Table 2.**
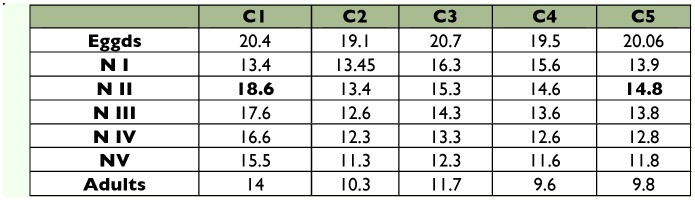
Life expectancy values obtained at the beginning of each stage of development for *Ronderosia bergi*, at 30° C and 14:10 L:D photoperiod.

Most curves of life expectancy based on age are usually decreasing (Rabinovich 1980). However, they frequently show certain maximums that demonstrate ages critical of the population in terms of mortality risks (Rabinovich 1980). In relation to this, it can be seen that the average life expectancy for the five cohorts peaked at approximately week 10 and slowly but steadily decreased until just past week 32 (Figure 2). Values of life expectancy registered at the beginning of each stage of development in each of the cohorts are detailed in Table 2.

The population parameters obtained are listed in Table 3. The net reproductive rate (R_0_) was 46.75 ± 11.2 with a minimum and a maximum of 28.9 and 83.7 respectively. The generational time (T) was 18.87 ± 1.67 with a minimum of 15 weeks for the cohort four and a maximum of 23.9 weeks for the cohort one. According to the parameter D (Duplication time), a population of *R. bergi* would require on average 3.31 ± 0.34 weeks to double in number. The intrinsic rate of population increase (r_m_) was 0.21 ± 0.021 with a minimum of 0.15 for cohort one and a maximum of 0.29 for the cohort four.

**Figure 3.**
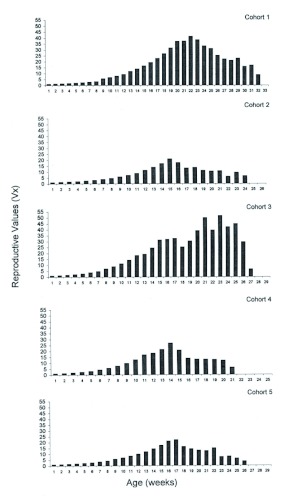
Reproductive values V_x_ of *Ronderosia bergi* at 30° C and 14:10 LD photoperiod. Unit time in weeks. High quality figures are available online.

**Table 3.**
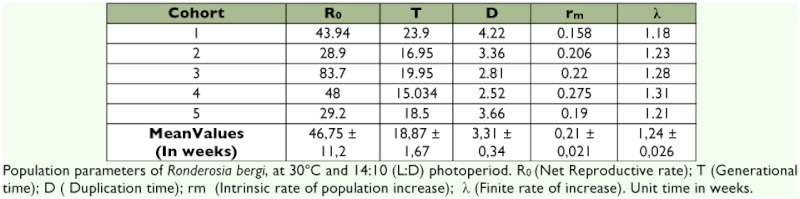
Population parameters of *Ronderosia* *bergi*, at 30° C and 14:10 LD photoperiod.

The finite rate of growth λ number of individuals added to the population per individual and per unit time was 1.24 ± 0.026 females per week with a minimum and a maximum of 1.18 and 1.31 individuals female per week respectively. The finite rate of growth followed a similar trend to the r_m_ while the duplication time was found to be inverse because the higher the r_m_ the shorter the time will be that a population requires for doubling its number. The mean reproductive values (V_x_) reached by each cohort were variable (Figure 3), and indicated that the highest contribution to the future population would occur between weeks 15 and 25. Cohort three had the highest value of V_x_ on week 23 with 51.42, and the mean highest value was 19.2 ± 9.6 also week 21.

When comparing these results with those coming from *R. bergi* of San Luis origin, there were no differences in the number of nymphal instars and the duration of each instar. Similarly, there were no significant differences in the longevity of adults among cohorts and gender (males and females) for each locality (Table 4). However, there were significant differences (H (1, N= 116) = 38.5 p= 0.000 (< 0.05)) regarding the longevity of adults between individuals from San Luis (135.25 ± 3.32 days) and individuals from Misiones (80.25 ± 2.38 days).

**Table 4.**
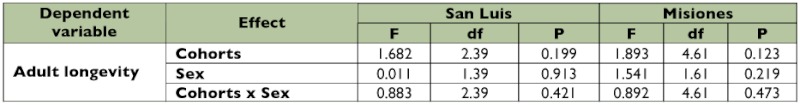
ANOVA of two- way performed in order to analize the variability in longevity of adults of *Ronderosia bergi* between cohorts and between males and females under controlled conditions for both studied localities.

## Discussion

Having facultative embryonic diapause is a salient trait of *R. bergi* that probably allows the species to go through unfavorable environmental conditions (Tauber et al. 1986; Danks 1987, 2007; Hunter 1997). Altitude and latitude related geographic variations normally generate environmental gradients that directly impact such a trait (Tanaka 1994; Burke et al. 2005; Danks 2006). It has been observed that geographic gradients tend to induce adaptive responses in the life cycles of acridomorph species with facultative embryonic diapause. For instance, geographic variation in photoperiod is an environmental factor with such a strong influence for diapause induction and life cycle in *Locusta migratoria* that, as demonstrated by Tanaka (1994), the proportion of bivoltine populations increases from north to south in Japan. Likewise, it was observed in *Melanoplus sanguinipes* that under shorter photoperiods the developmental time was reduced compared to longer photoperiods. Shorter days represent an indication of the growing season's end, and most insects respond by rushing their development, while the opposite is normally seen in higher latitudes where days are longer, representing for most insects an opportunity for continuous feeding (Fielding 2004). According to Gunawardene et al. (2004) it is important to consider that latitude derived variations in life cycles can be the result of adaptations to local climate. In this sense, longer life cycles are normally associated to areas of low feeding quality, cold environments, and/or unpredictable circum-stances (Danks 1992), whereas a shorter development time appears beneficial in unseasoned environments (Nylin and Gotthard 1998). Our results seem to agree with this because in Misiones province, where the climate is subtropical with a lack of clear seasonality (i.e., cold-hot or wet-dry), the life-span of individuals is shorter, and the general climatic conditions favor the development of an additional generation per year without the need of an embryonic diapause. On the contrary, individuals from San Luis province, where the climate is temperate with cold winters and hot summers, have a longer development time and one generation a year. Danks (1987) indicates that there are many species that have populations with obligate diapause in temperate regions and no diapause in tropical regions. More than one generation a year for *R. bergi* in Misiones appears to be in line with historical field observations by Liebermann (1948) in Santa Fé, another province in north eastern Argentina, where he has reported the presence of adults of *R. bergi* from December to July, too long a time frame for a single generation.

As it was mentioned earlier, *R. bergi* belongs to the Subfamily Melanoplinae, the melanoplines, which constitutes the largest grasshopper group, in terms of known species (N = 60), in Argentina (Cigliano and Lange 1998; Carbonell et al. 2006). Given that many melanopline species are frequently numerically dominant in grasshopper communities of different regions of the country, and several species constitute agricultural pests (COPR 1982; Cigliano et al. 2002; de Wysiecki et al. 2004; Lange et al. 2005), a comparison of the data obtained in the present study with those of other two melanopline species of wide distribution in Argentina, *Dichroplus elongatus* and *Dichroplus schulzi*, reared under the same conditions (de Wysiecki et al. 1997; Sánchez et al. 2001), seems appropriate. *Dichroplus elongatus* is frequently the most abundant species in grasshopper assemblages of the country, and has the widest geographic distribution, comprising almost the whole of the country (Cigliano and Lange 1999; Lange et al. 2005) (Table 5). *Ronderosia bergi* shows the highest median fecundity (125 ± 5.83 eggs/day) compared to 81.09 ± 14.02 eggs/day for *D. elongatus*, and 116.77 ± 32.60 eggs/day for *D. schulzi.* At present, the factors that seem to preclude *R. bergi* from reaching the levels of abundance usually seen in the field for *D. elongatus* (and to a lesser extent for *D. schulzi*) are not known. According to Hewitt (1985), the reproductive potential of acridids is the most important factor that determines the relative potential of the subsequent generation. Nevertheless, numerous biotic and abiotic factors (mainly temperature, precipitation, and food quality) affect the fecundity and the survival of these insects (Joern and Gaines 1990).

**Table 5.**
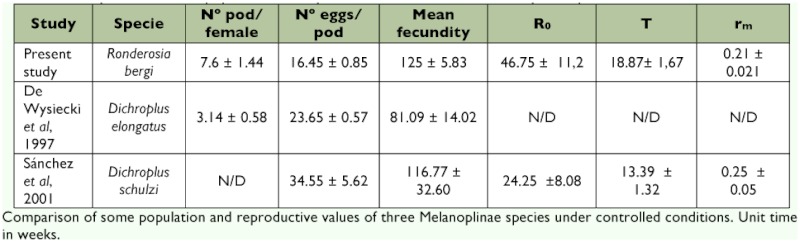
Comparison of some population and reproductive values of three Melanoplinae species under controlled conditions.

According to Rabinovich (1980), for discrete populations (i.e., no overlapping of generations), R_0_ is an appropriate parameter for estimating how much a population increases in each generation, and through this parameter, which is the rate of population growth. The mean R_0_ obtained for *R. bergi* shows a population increase per generation 1.92 times higher than *D. schulzi.*

As the intrinsic rate of natural growth, r is a genetically determined parameter that reflects the potential capacity of population multiplication (Rabinovich 1980). According to Price (1997), the best population parameter for the comparison of species is actually r_m_ because it takes into account the generation time, a parameter with much variability between species. Under certain conditions, the higher the r_m_, is, the higher the species capacity for reproduction and number is (Southwood 2000). Under the employed breeding conditions, it can be observed that even though *R. bergi* depicts a higher R_0_ than *D. schulzi*, the rate of increase is still lower than in the latter because *R. bergi* has a longer generation time.

Given that it may be assumed that the parameters estimated in this study were obtained at close to the best or ideal environmental conditions, and that such a scenario is unlikely to occur under natural conditions, field studies are envisaged in order to complement the observations reported here.
